# Efficacy of 3D-printed eye model to enhance retinoscopy skills

**DOI:** 10.1038/s41598-024-53321-8

**Published:** 2024-02-20

**Authors:** Dong Hyun Kim, Hee Kyung Yang, Changhoon Baek, Jongmo Seo, Jeong-Min Hwang

**Affiliations:** 1 Department of Ophthalmology, Seoul National University College of Medicine, Seoul National University Hospital, Seoul, Republic of Korea; 2 Department of Ophthalmology, Seoul National University College of Medicine, Seoul National University Bundang Hospital, Seongnam, Republic of Korea; 3 Department of Transdisciplinary Medicine, Seoul National University Hospital, Seoul, Republic of Korea; 4 Department of Electrical and Computer Engineering, Seoul National University College of Engineering, Seoul National University Hospital, Seoul, Republic of Korea

**Keywords:** Refractive errors, Experimental models of disease, Outcomes research, Physical examination, Health care economics

## Abstract

We conducted a prospective study to evaluate the efficacy of simulation-based education using a three-dimensional (3D)-printed schematic eye model in improving the retinoscopy refraction skills of medical students. A schematic eye model was printed using a fused deposition modeling-based 3D printer. Twenty medical students randomized into 3D (n = 10) and control (n = 10) groups received a 1-h lecture on the principles and methods of manifest refraction and were shown how to use the retinoscope and sciascope bars. The 3D group additionally attended a tutorial on the schematic eye. Both groups performed refractive examinations on four eyes of volunteer patients, and the results were recorded as a baseline. Instructor feedback and refraction practice was provided with the 3D group or with control group. To account for subject fatigue, patients spent no more than 8 min on the examination. After a 1-h break to allow for fatigue and familiarity, refraction tests were repeated on four randomly selected eyes of patients. Students’ refraction readings were compared with the autorefractor values using a spherical equivalent value and blur strength. All participants measured the time required to complete the refraction test and reported their subjective confidence in the results of each refraction test. Refractive errors before and after training did not differ between the control and 3D groups, with a significant improvement in errors observed in both groups (p = 0.005 and 0.008, respectively). The time to complete refraction before and after training did not differ between the two groups, both of which showed a significant reduction in time (p = 0.005 and 0.028, respectively). Pre- and post-training confidence scores for the accuracy of each refraction on a 10-point Likert scale were not significantly different. However, when comparing score changes between pre- and post-training, only the control group showed a significant increase in confidence (p = 0.005). Tests for the non-inferiority of refractive errors after training indicated that the 3D group was non-inferior to the control group. In conclusion, training in retinoscopy refraction skills using a 3D-printed eye model resulted in significant improvement in accuracy and speed compared to practice with real patients. Except for better confidence in the control group, schematic eye model training was not inferior to practice with real patients.

## Introduction

Retinoscopy is an objective tool to assess refractive errors^1^. In the field of ophthalmology, the retinoscopic examination may be the most important and indispensable test for providing the best-corrected visual acuity^2^. Despite advances in automated refractors, objective manifest refraction remains essential, especially for patients with underlying diseases that cause media opacities, such as cataract or corneal opacity^3^. In addition, the objective manifest refraction test is particularly important in pediatric ophthalmology, as young children may have difficulty cooperating with automated refraction measurements^4^. As a result, the objective manifest refraction test is essential to accurately determine refractive errors in these cases and ensure the appropriate prescription for visual correction^5^.

Practicing retinoscopic examinations is crucial for ophthalmologists, optometrists, and orthoptists. Repeated practice and experience are essential to improve refraction accuracy^6,7^. Traditional methods involving repeated testing on patients or standardized actors are very effective, but have limitations such as patient fatigue, recruitment difficulties, and potential risks to real patients^8^. The development of schematic mechanical eyes may be a valuable solution that addresses these challenges^9,10^. However, many advanced simulators on the market can be prohibitively expensive, limiting access for institutions with limited resources^11^.

Many commercial schematic eye models have been developed for training in retinoscopic examinations. In the era of three-dimensional (3D) printing technology, a schematic eye could be made at a much lower price^12^. In our previous study, we presented a schematic eye that can be printed in less than 4 h for under 5 USD on a 3D printer of the fused deposition modeling type, the most common type printer^12^. The device was designed to facilitate the adjustment of refractive errors by inserting a trial lens. If this type of inexpensive schematic eye could enhance retinoscopic skills, it would be helpful in improving healthcare worldwide. We performed this study to assess the efficacy of simulation-based education using a 3D printed-schematic eye model in enhancing the retinoscopic skills of medical students.

## Results

The mean age of students in the control group was 24.0 ± 2.6 years of age and in the 3D group was 23.3 ± 2.8 years of age, which was not significantly different (p = 0.579). The male-to-female ratio was 7:3 in both groups.

The patients who volunteered for the study were between the ages of 19 and 34, with a mean age of 23.5 ± 2.7 years for the control group and 24.4 ± 3.9 years for the 3D group with no significant difference (p = 0.816). Spherical powers were − 2.28 ± 3.20 D in the control group and − 2.53 ± 2.53 D in the 3D group, and cylindrical powers were − 1.13 ± 0.73 in the control group and − 0.81 ± 0.61 in the 3D group, both of which were not statistically different (p = 0.545, 0.130, respectively). In the control group, the cylindrical axis consisted of 16 eyes of with-the-rule (WTR) astigmatism and 4 eyes of oblique axis. Meanwhile, in the 3D group, there were 12 eyes of WTR astigmatism, 12 eyes of against-the-rule (ATR) astigmatism, and 4 eyes of oblique axis (Table 1).

**Table 1:** Characteristics of the volunteer patients on whom students performed refractions.

	Control	3D group	p value
Age (year)	23.5 ± 2.7 (19 ~ 27)	24.4 ± 3.9 (20 to 34)	0.816
Spherical (D)	− 2.28 ± 3.20 (− 7.25 to + 1.00)	− 2.53 ± 2.53 (− 8.00 to + 0.50)	0.545
Cylindrical (D)	− 1.13 ± 0.73 (− 2.75 to − 0.25)	− 0.81 ± 0.61 (− 2.50 to − 0.25)	0.130
Axis (WTR:ATR:Oblique)	16:0:4	12:4:4	

Data are mean ± standard deviation values (minimum–maximum).

p value determined by Mann–Whitney test.

### Spherical equivalent refraction results

**Figure 1:**
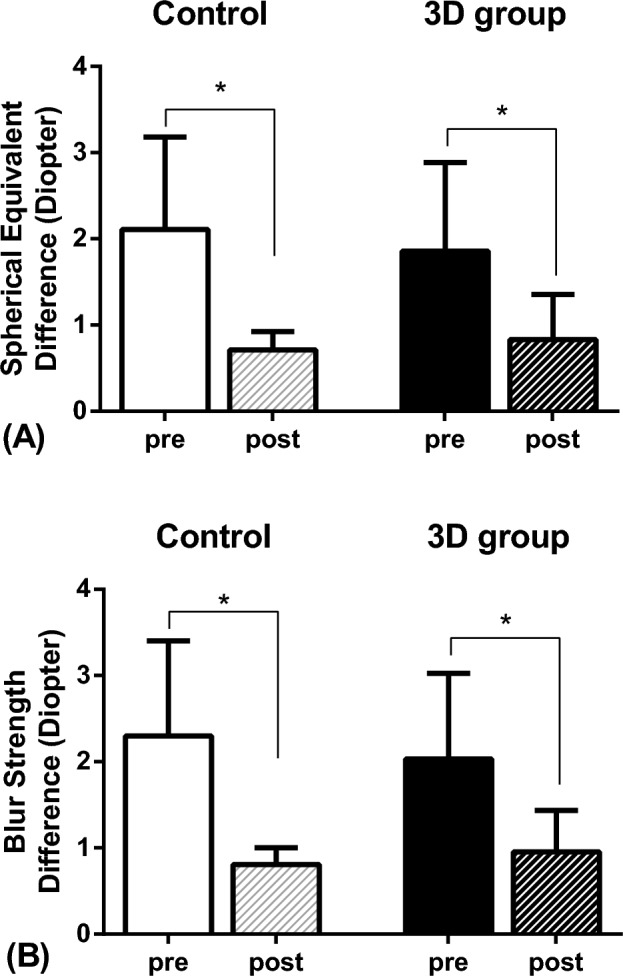
(**A**) The difference in spherical equivalent compared to an autorefractometer. No significant difference was observed in the Mann–Whitney test between the two groups before and after training. Both the control and 3D groups showed a significant improvement in accuracy after training (p = 0.005 and p = 0.008, respectively, Wilcoxon signed rank test). (**B**) The difference in blur strength compared to an autorefractometer. No significant difference was observed in the Mann–Whitney test between the two groups before and after training. Both the control and 3D groups showed a significant improvement in accuracy after training (p = 0.005 and p = 0.017, respectively, Wilcoxon signed rank test).

Before training, the students’ refractive error (spherical equivalents) compared with that of the autorefractor was 2.11 ± 1.08 D in the control group and 1.86 ± 1.03 D in the 3D group, with no statistically significant difference observed between the two groups (p = 0.604). After training, the refractive error was 0.71 ± 0.21 D in the control group and 0.83 ± 0.52 D in the 3D group, with no significant difference (p = 0.796). A significant improvement in refractive error was observed in both the control group and 3D group (p = 0.005 and 0.008, respectively) (Fig. 1A).

### Vector analysis

The power vector analysis before and after training is presented in Table 2. Before training, the students’ blur strength compared with that of the autorefractor was 2.30 ± 1.10 D in the control group and 1.83 ± 1.13 D in the 3D group, with no statistically significant difference observed between the two groups (p = 0.353). After training, the blur strength was 0.81 ± 0.19 D in the control group and 0.95 ± 0.48 D in the 3D group, with no significant difference (p = 0.739). A significant improvement in blur strength was observed in both the control group and 3D group (p = 0.007 and 0.017, respectively) (Fig. 1B).

**Table 2:** Summary of the mean value and standard deviation of power vector analysis before and after training.

	Pre training	Post training	p value
Control group			
M	2.11 ± 1.08	0.71 ± 0.21	0.005
J_0_	0.79 ± 0.47	0.34 ± 0.08	0.005
J_45_	0.26 ± 0.06	0.15 ± 0.04	0.007
B	2.30 ± 1.10	0.81 ± 0.19	0.005
3D group			
M	1.86 ± 1.03	0.83 ± 0.52	0.008
J_0_	0.69 ± 0.37	0.35 ± 0.23	0.012
J_45_	0.18 ± 0.04	0.10 ± 0.04	0.007
B	1.83 ± 1.13	0.95 ± 0.48	0.017

p value determined by Wilcoxon signed rank test.

### Time spent

**Figure 2:**
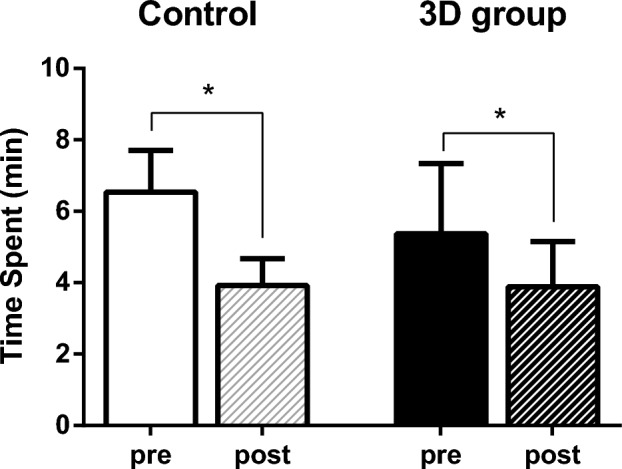
Time spent for refraction exam. Both the control and 3D groups showed significant improvement in examination time after training (p = 0.005 and p = 0.028, respectively).

The time to complete the refraction test was 6.5 ± 1.2 min in the control group and 5.4 ± 2.0 min in the 3D group, with no significant difference at pre-training (p = 0.313). After training, there was no significant difference between the two groups (3.9 ± 0.8 min in control group and 3.9 ± 1.3 min in the 3D group; p = 0.875). Comparing pre- and post-training, both the control and 3D groups showed a significant reduction in time (p = 0.005 and 0.028, respectively) (Fig. 2).

### Confidence level

**Figure 3:**
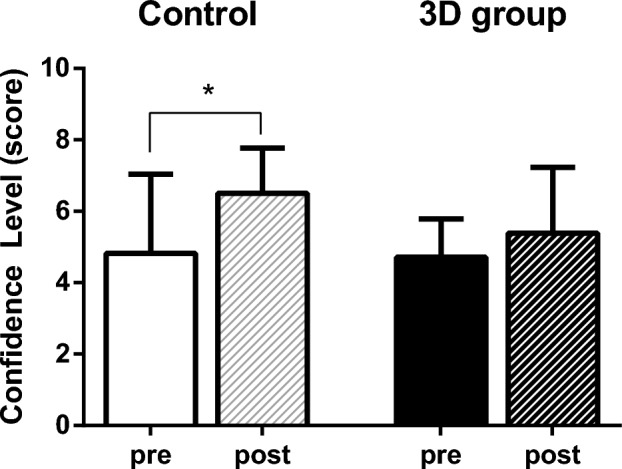
Confidence level. No significant difference was observed between the two groups in the Mann–Whitney test before training (p = 0.684) and after training (p = 0.143). When comparing pre- and post-training using the Wilcoxon rank test, the control group showed a significant improvement in confidence (p = 0.005), while no significant difference was observed in the 3D group (p = 0.083).

When asked to rate their pre-training confidence in the accuracy of each refraction on a 10-point Likert scale, the control group scored 4.9 ± 2.2 and the 3D group scored 4.3 ± 1.8, which was not significantly different (p = 0.684). After training, the control group scored 6.5 ± 1.3 and the 3D group scored 5.4 ± 1.8, which was not significantly different (p = 0.143). Comparing pre- and post-training, the 3D group showed no significant change in confidence scores (p = 0.083), whereas the control group showed a significant increase in confidence (p = 0.005) (Fig. 3).

### Test for non-inferiority of refraction errors

**Figure 4:**
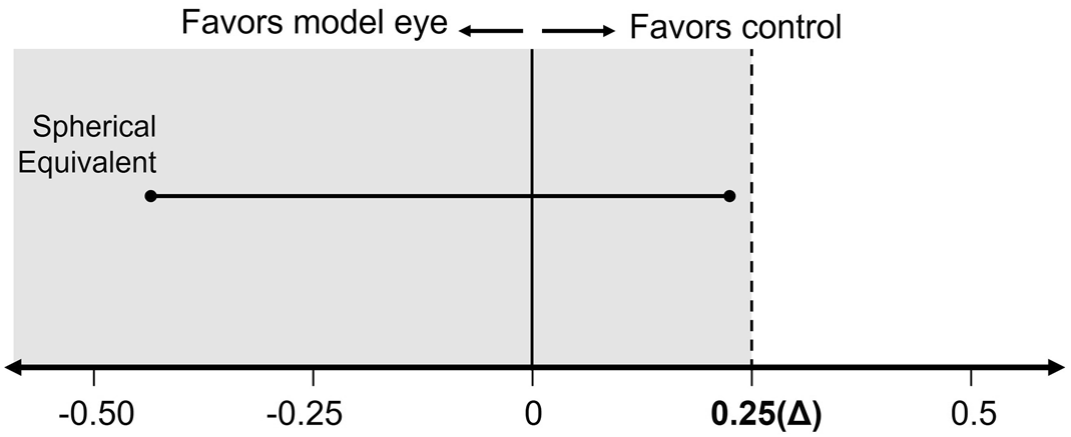
Differences in autorefractor spherical equivalent gap between the 3D group and the real patient group. The circled bar indicates a 95.7% confidence interval range. The vertical solid line indicates an intersection through 0. The dotted line indicates an intersection through 0.25, which was defined as the non-inferiority margin of the 3D group to the real patient group.

The approximate 95% confidence interval (exact level is 95.7%) of refractive errors in spherical equivalent values between the 3D group and control group was − 0.43 D to 0.23 D. Defining the non-inferiority margin as Δ0.25 D, a clinically relevant refractive error, the 95% confidence interval for the errors in spherical equivalents between the two groups did not exceed Δ0.25 D, indicating that the 3D group was non-inferior to the control group (Fig. 4).

## Discussion

This study showed that novice medical students comparatively improved their retinoscopic refraction skills either with a schematic eye or with real patients. Both groups showed a significant improvement in errors after training and significantly reduced time to complete the refraction test. Although tests for the non-inferiority of spherical equivalents after training indicated that the 3D group was non-inferior to the control group, only the control group showed a significant increase in confidence on a 10-point Likert scale. This finding is consistent with previous studies in healthcare that reported that real-patient cases were more effective in improving students’ refraction skills than digital and paper cases^13^.

Three-dimensional printing technology has been widely applied in the field of ophthalmology, including ocular prostheses, orbital implants, drug delivery systems, retrobulbar-anesthesia simulators^12,14–17^. However, there has been no report of a schematic eye except ours^12^. In our previous study, we made this schematic eye for less than $5 using a 3D printer^12^. This price is competitive to any of the available commercial schematic eyes. This type of inexpensive and convenient schematic eye could open a new era of simulation as an educational tool.

Retinoscopy is an indispensable tool in the field of eye care, and ophthalmologists, optometrists, and orthoptists should master their skills. Therefore, education on retinoscopic refraction could be essential for training in the field of eye care. Our schematic eye was helpful in the practice of retinoscopy and successfully enhanced retinoscopic skills. Traditional methods of retinoscopy training involve repeated testing of patients or standardized actors, but these methods lead to subject fatigue and recruitment difficulties^18,19^. Other studies have reported a web-based learning environment that simulates a clinical subjective refraction assessment, a virtual refractor, or a psychophysical technique for estimating the accuracy and precision of manifest refraction, all of which have found simulation to be beneficial for training^20–22^. In this study, the 3D-printed eye model demonstrated non-inferior improvements in refractive accuracy compared to students practicing on real patients, demonstrating its potential as an effective refractive training tool, similar to other simulators.

In our study, the group that practiced with the 3D-printed eye model showed significant improvements in refractive accuracy and time but less improvement in their perceived confidence in refraction. Simulation training provides a learning environment for managing complex crisis situations without compromising the safety of human patients^23^. Repeated simulations increase student confidence^24^. In our research, the simulation training lasted only one hour, which may explain the smaller confidence gains than those of the control group that practiced on real patients. Repeated practice was expected to contribute to confidence gains, as well as improvements in refractive accuracy and time.

The high cost of simulators has been a commonly cited critique of simulation training^25^. This is because of the significant importance of assessing the cost-effectiveness and cost–benefit of simulator training^26^. Currently, several mechanical schematic eyes are available within the range of 60 to 120 USD, but the price can impede student access. In our study, we printed a refraction-training schematic eye model in less than 4 h using a 3D printer at a cost of approximately $0.92 to $1.24. Thus, our results indicated that this device could offer affordable training by reducing costs.

This study had some limitations. First, we only included medical students. We could not perform the same study with optometry students because we do not have a system for optometrists in Korea. Second, no other studies have compared training using a schematic eye model versus real patients. Therefore, we cannot compare our results with those of previous studies. Third, medical students who want to acquire refraction skills could be highly motivated to learn more, thus affecting their results. Finally, this study only assessed the immediate effects of training, and further follow-up would be warranted to determine the long-term retention of skills and knowledge acquired through 3D-printed eye model training. To strengthen the evidence of the efficacy and feasibility of 3D training aids in the field of refraction, future studies should consider larger and more diverse samples, longer training durations, and more comprehensive comparisons with other training methods, especially with commercial retinoscopy simulators.

In conclusion, retinoscopic refraction training using a 3D-printed schematic eye model resulted in a significant improvement in errors and a reduction in time to complete the refraction, similar to the practice with real patients. Except for a difference in confidence in the control group, schematic eye training was not inferior to practice with actual patients.

## Methods

### 3D printed-schematic eye model

**Figure 5:**
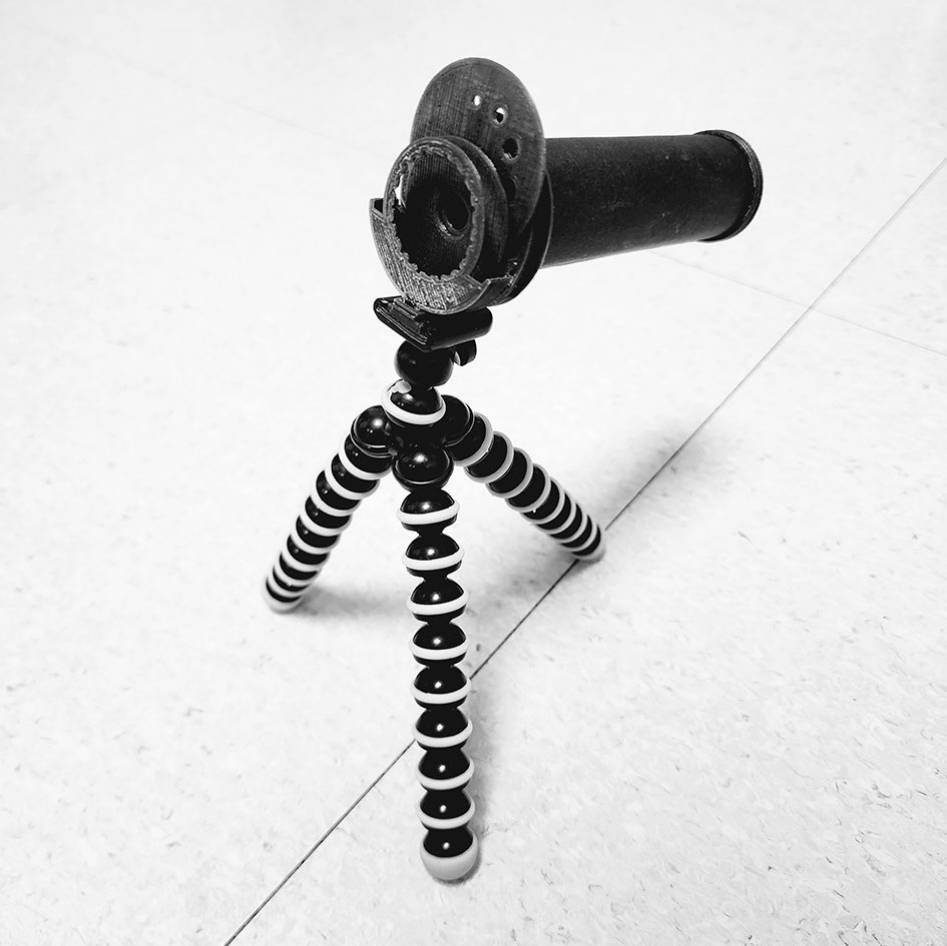
3D printed schematic eye. Trial lenses can be mounted on the front to achieve the desired refractive error, and a disc with holes of different sizes can be rotated to vary the size of the pupil. The body tube is 10 cm long and has a default refractive error of + 10 D.

A schematic eye model for retinoscopy training was designed and printed using a fused deposition modeling-based 3D printer as described in a previous study^12^. Seven parts were printed and assembled with additional components of a paper cylinder, + 10-diopter (D) spherical lens, and tripod (Fig. 5). The entire printing process required less than one hour to complete using a conventional 3D printer.

### Participants

Twenty medical students participated in this study. Participation was voluntary, and written informed consent, which could be withdrawn at any time without penalty, was obtained from each student before the study began. The participants were recruited through an IRB-approved advertisement and informed that participation in the study was not tied to their grades. The study was conducted outside of the grading period, and the researcher did not have access to the data collected until the class grades were posted. The study protocol adhered to the tenets of the Declaration of Helsinki and was approved by the Institutional Review Board of Seoul National University Bundang Hospital (B-2201-735-305).

All students received a 1-h lecture on the principles and methods of manifest refraction and were shown how to use the retinoscope and sciascope bars. The students were randomized into two groups: a 3D-printed schematic eye training aid group (3D group, n = 10) and a real patient group (control group, n = 10). The 3D group additionally attended a tutorial on the schematic eye.

Based on the previous lecture, the students in both groups performed refractive examinations on four eyes of the volunteer patients and recorded the results as a baseline. After the baseline examination, 1 h of instructor feedback and refraction practice were provided. The instructor provided personalized feedback with practice repetition for more eyes or theoretical reinforcement based on the student’s individual needs. The 3D group practiced with the 3D-printed schematic eye training device, whereas the control group practiced with volunteer patients. To account for subject fatigue, patients were not allowed to spend more than 8 min on the examination. After a 1-h break to allow for fatigue and familiarity, refraction tests were repeated on four eyes of randomly selected patients. The students’ refractive error results were compared with those of the autorefractor by taking the spherical equivalent value and blur strength (mean of three readings on a Huvitz HRK-8000A; Anyang, Korea).

All participants measured the time taken to complete the refraction test and provided their subjective confidence in the results of each refraction test (on a 10-point Likert scale: 10 = very accurate, 1 = not at all accurate).

### Definitions

The orientation of astigmatism was defined as with-the-rule (WTR) astigmatism in case of the axis of the minus cylinder is placed between 0 and 30 and 150 and 180 degrees, oblique astigmatism between 30 and 60 and 120 and 150 degrees, and against-the-rule (ATR) astigmatism between 60 and 120 degrees^27^.

To conduct the vectorial analysis, we transformed the refractive astigmatism from spherocylinder notation to power vector notation using Fourier transformation with the following equations (S (spherical diopters), C (cylindrical diopters), α (axis) B (blur strength))^28^.

$$M=S+\frac{C}{2},$$

$${J}_{0}=-\frac{C}{2}\times {\text{cos}}2\alpha ,$$

$${J}_{45}=-\frac{C}{2}\times {\text{sin}}2\alpha ,$$

$$B=\sqrt{{M}^{2}+{{J}_{0}}^{2}+{{J}_{45}}^{2}}.$$

### Statistical analysis

Data were analyzed using SPSS version 26.0 (IBM SPSS Statistics for Windows, V.26.0; IBM Corp, Armonk, New York, USA). Results were considered statistically significant at p < 0.05. Data are presented as mean ± standard deviation unless otherwise noted. Mann–Whitney tests were used for comparisons between the two groups, and Wilcoxon signed-rank tests were used for comparisons before and after training. Confidence intervals and non-inferiority tests for nonparametric tests were conducted according to a previous study^29^.

### Ethics statement

Ethics approval was obtained from our institutional ethics review board.

## Data Availability

The Institutional Review Board of Seoul National University Bundang Hospital/Ethics committee has placed ethical restrictions to protect patient identities. However, the data are available to anyone who is interested without restriction. The minimal data set will be available upon request. For data requests, please contact the SNUBH IRB office at 82-31-787-8804, 98614@snubh.org.

## References

[1] Akil H, Keskin S, Cavdarli C (2015). Comparison of the refractive measurements with hand-held autorefractometer, table-mounted autorefractometer and cycloplegic retinoscopy in children. Korean J. Ophthalmol..

[2] Yoo SG, Cho MJ, Kim US, Baek SH (2017). Cycloplegic refraction in hyperopic children: Effectiveness of a 0.5% tropicamide and 0.5% phenylephrine addition to 1% cyclopentolate regimen. Korean J. Ophthalmol..

[3] Heher KL, Stark WJ, Miller NR (1993). Oil-drop cataracts. J. Cataract Refract. Surg..

[4] Dahlmann-Noor AH, Vivian AJ (2008). A comparison of photorefraction and retinoscopy in children. J. AAPOS.

[5] Hollis J, Allen PM, Heywood J (2022). Learning retinoscopy: A journey through problem space. Ophthal. Physiol. Opt..

[6] Zadnik K, Mutti DO, Adams AJ (1992). The repeatability of measurement of the ocular components. Investig. Ophthalmol. Vis. Sci..

[7] Walline JJ, Kinney KA, Zadnik K, Mutti DO (1999). Repeatability and validity of astigmatism measurements. J. Refract. Surg..

[8] Aydin P, Gunalp I, Hasanreisoglu B, Unal M, Erol Turacli M (2006). A pilot study of the use of objective structural clinical examinations for the assessment of ophthalmology education. Eur. J. Ophthalmol..

[9] Dodaro NR, Maxwell DP (1995). An eye for an eye. A simplified model for teaching. Arch. Ophthalmol..

[10] Lewallen S (2006). A simple model for teaching indirect ophthalmoscopy. Br. J. Ophthalmol..

[11] Donovan L, Brian G, du Toit R (2008). A device to aid the teaching of retinoscopy in low-resource countries. Br. J. Ophthalmol..

[12] Baek C, Seo J-M (2016). Development of schematic eye for retinoscopy training using 3D printer. Ann. Optom. Contact Lens.

[13] Li J (2013). Comparison of three problem-based learning conditions (real patients, digital and paper) with lecture-based learning in a dermatology course: A prospective randomized study from China. Med. Teach..

[14] Kang S (2018). Generation of customized orbital implant templates using 3-dimensional printing for orbital wall reconstruction. Eye (Lond.).

[15] Kim BR (2021). A pilot clinical study of ocular prosthesis fabricated by three-dimensional printing and sublimation technique. Korean J. Ophthalmol..

[16] Park BC, Kim HT, Koh JW (2021). New biodegradable drug delivery system for patients with dry eye. Korean J. Ophthalmol..

[17] Choi YJ, Joo YH, Oh BL, Lee JC (2021). 3D-printed ophthalmic-retrobulbar-anesthesia simulator: Mimicking anatomical structures and providing tactile sensations. IEEE J. Transl. Eng. Health Med..

[18] Rodriguez-Lopez, V. & Dorronsoro, C. Beyond traditional subjective refraction. *Curr. Opin. Ophthalmol.* **33**, 228–234, 10.1097/ICU.0000000000000834 (2022). 10.1097/ICU.0000000000000834 35102097

[19] Levine, N. R. Improving student understanding and management of patients through role-playing and video taping. *Am. J. Optom. Physiol. Opt.* **53**, 95–99, 10.1097/00006324-197602000-00009 (1976). 10.1097/00006324-197602000-00009 937479

[20] Woodman-Pieterse, E. C., De Souza, N. J. & Vincent, S. J. The influence of a novel simulated learning environment upon student clinical subjective refraction performance: A pilot study. *Clin. Exp. Optom.* **99**, 342–349. 10.1111/cxo.12374 (2016). 10.1111/cxo.12374 27001687

[21] Alhazmi, M. S., Butler, C. W. & Junghans, B. M. Does the virtual refractor patient-simulator improve student competency when refracting in the consulting room? *Clin. Exp. Optom.* **101**, 771–777. 10.1111/cxo.12800 (2018). 10.1111/cxo.12800 29895093

[22] Bharadwaj, S. R., Malavita, M. & Jayaraj, J. A psychophysical technique for estimating the accuracy and precision of retinoscopy. *Clin. Exp. Optom.* **97**, 164–170, 10.1111/cxo.12112 (2014). 10.1111/cxo.12112 24117784

[23] Mitchell, M., Newall, F., Sokol, J., Heywood, M. & Williams, K. Simulation-based education to promote confidence in managing clinical aggression at a paediatric hospital. *Adv. Simul. (Lond)* **5**, 21. 10.1186/s41077-020-00139-9 (2020). 10.1186/s41077-020-00139-9 PMC7425032 32817808

[24] McInerney, N., Nally, D., Khan, M. F., Heneghan, H. & Cahill, R. A. Performance effects of simulation training for medical students—a systematic review. *GMS. J. Med. Educ.* **39**, Doc51. 10.3205/zma001572 (2022). 10.3205/zma001572 PMC9733478 36540561

[25] Zendejas, B., Wang, A. T., Brydges, R., Hamstra, S. J. & Cook, D. A. Cost: The missing outcome in simulation-based medical education research: A systematic review. *Surgery* **153**, 160–176. 10.1016/j.surg.2012.06.025 (2013). 10.1016/j.surg.2012.06.025 22884087

[26] Maloney, S. & Haines, T. Issues of cost-benefit and cost-effectiveness for simulation in health professions education. *Adv. Simul. (Lond)* **1**, 13, 10.1186/s41077-016-0020-3 (2016). 10.1186/s41077-016-0020-3 PMC5806357 29449982

[27] Nemeth, G., Szalai, E., Berta, A. & Modis, L., Jr. Astigmatism prevalence and biometric analysis in normal population. *Eur. J. Ophthalmol.* **23**, 779–783. 10.5301/ejo.5000294 (2013). 10.5301/ejo.5000294 23640506

[28] Thibos, L. N., Wheeler, W. & Horner, D. Power vectors: an application of Fourier analysis to the description and statistical analysis of refractive error. *Optom. Vis. Sci.* **74**, 367–375. 10.1097/00006324-199706000-00019 (1997). 10.1097/00006324-199706000-00019 9255814

[29] Campbell, M. J. & Gardner, M. J. Calculating confidence intervals for some non-parametric analyses. *Br. Med. J.* (Clin Res Ed) **296**, 1454–1456. 10.1136/bmj.296.6634.1454 (1988). 10.1136/bmj.296.6634.1454 PMC2545906 3132290

